# G-quadruplex aptamer targeting Protein A and its capability to detect *Staphylococcus aureus* demonstrated by ELONA

**DOI:** 10.1038/srep33812

**Published:** 2016-09-21

**Authors:** Regina Stoltenburg, Petra Krafčiková, Viktor Víglaský, Beate Strehlitz

**Affiliations:** 1 UFZ - Helmholtz Centre for Environmental Research, Department of Soil Ecology, Theodor-Lieser-Straße 4, 06120 Halle, Germany; 2 P.J.Šafárik University, Faculty of Sciences, Institute of Chemistry, Dept. of Biochemistry, Moyzesova 11, 04011 Košice, Slovakia; 3 UFZ - Helmholtz Centre for Environmental Research, Department Environmental and Biotechnology Centre, Permoserstraße 15, 04318 Leipzig, Germany

## Abstract

Aptamers for whole cell detection are selected mostly by the Cell-SELEX procedure. Alternatively, the use of specific cell surface epitopes as target during aptamer selections allows the development of aptamers with ability to bind whole cells. In this study, we integrated a formerly selected Protein A-binding aptamer PA#2/8 in an assay format called ELONA (Enzyme-Linked OligoNucleotide Assay) and evaluated the ability of the aptamer to recognise and bind to *Staphylococcus aureus* presenting Protein A on the cell surface. The full-length aptamer and one of its truncated variants could be demonstrated to specifically bind to Protein A-expressing intact cells of *S. aureus*, and thus have the potential to expand the portfolio of aptamers that can act as an analytical agent for the specific recognition and rapid detection of the bacterial pathogen. The functionality of the aptamer was found to be based on a very complex, but also highly variable structure. Two structural key elements were identified. The aptamer sequence contains several G-clusters allowing folding into a G-quadruplex structure with the potential of dimeric and multimeric assembly. An inverted repeat able to form an imperfect stem-loop at the 5′-end also contributes essentially to the aptameric function.

The enzyme-linked immunosorbent assay (ELISA) is a fundamental tool of immunological, medical, and biochemical research, and was firstly developed in 1971 as a replacement for the radioimmunoassay (RIA)^1^. ELISA is based on the principle of antigen-antibody interactions in combination with photometric visualisation of the binding results and is typically performed in microtiter plates. It aims at the detection and quantification of very small quantities of antigens such as proteins, peptides, hormones, or antibodies in a liquid sample.

With the emergence of novel molecular recognition elements, termed aptamers, their potential to replace or complement the role of antibodies in the classical ELISA was tested resulting in the development of ELONA (Enzyme-Linked OligoNucleotide Assay) and was first reported by Drolet *et al*. in ref. 2. Aptamers are artificial, short single-stranded nucleic acid molecules with particular properties comparable to those of antibodies^3^,^4^. Firstly described in the 1990s, they have become a very attractive class of targeting molecules^5^,^6^. Aptamers are obtained by an iterative *in vitro* selection and amplification method called SELEX, Systematic Evolution of Ligands by Exponential Enrichment^7^,^8^,^9^, and they are selected for the highly affine and specific recognition and binding to target molecules like small organic compounds, single peptides and proteins, or complex structures and whole cells^10^. Their functionality is based on a complex three-dimensional structure formed by intramolecular folding in accordance with the primary sequence of the aptamers.

Aptamers have been verified to be applicable as analytical agents in a variety of biosensors (aptasensors) and detection assays^11^,^12^,^13^ including ELONA as one of them^14^,^15^,^16^,^17^. Different ELONA configurations (Fig. 1) derived from ELISA have been described^18^, in which aptamers were used either in combination with antibodies or by replacing them completely. Variations and optimisations of the different ELONA formats often concern the immobilisation procedure of the target molecules or the aptamers themselves on the surface, the enzyme-substrate combination for signal generation, and assay modifications aiming at signal amplification for an increased sensitivity^18^,^19^,^20^.

In this work, we applied a recently selected aptamer for Protein A in ELONA to evaluate its ability to recognise and bind to its target protein in the whole cell context of *Staphylococcus aureus*^21^. Protein A is a typical component of the cell wall of this gram-positive bacterium and also represents one of its virulence factors. *S. aureus* is a ubiquitous human pathogen causing a broad range of infections from minor skin infections to systemic and life-threatening diseases such as pneumonia, meningitis, osteomyelitis, toxic shock syndrome (TSS), and sepsis^22^,^23^. In particular the antibiotic-resistant strains (MRSA: methicillin-resistant *S. aureus*) represent a major public health problem. Located on the cell surface, Protein A represents a very suitable target for analytical agents to detect the bacterial pathogen. There is a growing need for rapid, cost-efficient methods to detect bacterial infections and contaminations in food, feed, or water including the use of alternative recognition elements such as aptamers. Several aptamers have been used for the detection of specific bacterial pathogens including *E. coli*, *Salmonella*, *Campylobacter*, *Staphylococcus*, or *Listeria*^24^,^25^.

The functionality of each aptamer is importantly defined by its folding into a characteristic three-dimensional shape. Knowledge about the sequence region and structural elements of the aptamer responsible for target binding would help to design an efficient aptamer-based assay. In our previous work, we started to localise the relevant sequence regions by truncation experiments. We also described some G-rich parts in the sequence of the Protein A-binding aptamer supposing that it could form a typical G-quadruplex structure^21^. In order to elucidate this potential structural feature, we additionally applied circular dichroism (CD) spectroscopy and native gel electrophoresis in this study. G-quadruplexes (G4) represent a special tertiary conformation of single-stranded DNA and RNA. They are characterised by two or more planar G-quartets stacked on top of each other. But they are also known as highly polymorphic molecules with the potential to form high-ordered multimeric structures^26^,^27^,^28^. G-quadruplex forming aptamers have been repeatedly obtained mainly for proteins but also for small molecule targets during *in vitro* selection procedures indicating that G-quadruplexes belong to the most common structures of aptamers^29^,^30^.

## Results and Discussion

### Protein A-binding aptamer PA#2/8 applied in ELONA

An aptamer-based ELONA was established to prove the functionality of the previously selected aptamer PA#2/8 for Protein A of *S. aureus*^21^ under the specific assay design. ELONA was performed by immobilisation of native or recombinant Protein A in microtiter plates followed by addition of 3′-biotinylated aptamer PA#2/8 for binding. As shown in Fig. 2, this assay setup works very well with the applied aptamer-target-system. The binding features of PA#2/8 obtained in former studies^21^ by using bead-based fluorescence binding assays, SPR experiments (surface plasmon resonance), or MST experiments (MicroScale Thermophoresis) were reproduced by ELONA. Aptamer PA#2/8 strongly binds to native and recombinant Protein A (Fig. 2). It clearly distinguishes its target from functional related proteins like Protein G and Protein L, which were not bound. The aptamer also does not bind to human serum albumin (HSA) used as unspecific control protein. In contrast to the aptamer, the unselected 3′-biotinylated SELEX library (BANK-C, see Table 1) was not able to bind to Protein A. BANK-C represents the starting random oligonucleotide library from which the aptamer PA#2/8 was selected for binding to Protein A by the FluMag-SELEX process as described previously^21^. The library consists of a multitude of different oligonucleotides comprising large sequence diversity. In downstream binding analyses with selected aptamers like ELONA in this study, the library was usually used as negative control. All measured signals from negative reactions were in the range of the blank signal, which was produced by a reaction without any immobilised protein.

Recent binding studies revealed that the 5′-end of aptamer PA#2/8 is very important for its functionality^21^. Deletion of nucleotides (Supplementary Fig. S1) or blocking of the 5′-end, e.g., due to immobilisation of the aptamer over this side on a surface, result in a strongly reduced or loss of binding ability of the aptamer to Protein A. The ELONA setup is specified by coupling of the avidin-peroxidase-conjugate to the biotinylated aptamer bound to the immobilised target. The efficiency of this coupling could be influenced differently in dependence of the biotinylation site of the aptamer (5′- or 3′-end), and could also destabilise the aptamer-target complex resulting in detaching of the aptamer. Therefore, the 5′- or 3′-biotinylated aptamers were tested. Both biotinylated variants of PA#2/8 were functional in ELONA (Fig. 3). A slightly higher binding signal was measured for the 3′-biotinylated aptamer in comparison to the 5′-biotinylated form.

As an intact 5′-end is important, the aptamer can be truncated at its 3′-end to a certain extent without loss of function. This could be shown for three truncations of different lengths (Table 1) using bead-based fluorescence binding assays (Supplementary Fig. S1). Two 3′-truncated variants PA#2/8[S1-58] and PA#2/8[S1-43] were found to have even a better binding ability to Protein A in comparison to the full-length aptamer, which is in addition supported by MST experiments^21^ analysing the binding affinity. In contrast, the variant PA#2/8[S1-50] shows a reduced binding behaviour to Protein A. Interestingly, although all three truncated aptamer variants were functional, only PA#2/8[S1-58] works very well in ELONA (Fig. 3). A differentiation in binding signal depending on the biotinylation site of this aptamer variant could not be observed. The binding signals of PA#2/8[S1-50] and PA#2/8[S1-43] were in the range of those of the library representing the negative control.

### Evaluation of binding affinity of aptamer PA#2/8 and PA#2/8[S1-58] using ELONA

ELONA also allows the evaluation of the binding affinity of the full-length aptamer PA#2/8 and its truncated variant PA#2/8[S1-58] to Protein A. The dissociation constants (K_D_) were calculated from binding data using a concentration series of 5′- or 3′-biotinylated aptamers in the range of 10–3,000 nM added to Protein A-coated wells (rec. Protein A, P7837). K_D_-values were obtained in the low nanomolar range for all tested aptamer variants (Fig. 4). The highest affinity with K_D_ = 11.3 ± 1.4 nM was determined for the truncated 3′-biotinylated aptamer PA#2/8[S1-58], followed by its 5′-biotinylated variant with K_D_ = 23.7 ± 2.0 nM. Significant lower affinity was calculated for the full-length aptamer PA#2/8, whereas again the 3′-biotinylated variant resulted in a better K_D_ of 101.4 ± 5.9 nM than the 5′-biotinylated variant with a K_D_ of 189.9 ± 13.0 nM.

In previous studies, the affinity of the Protein A-binding aptamer was measured using different assays either with immobilisation of one of the binding partners (fluorescent bead-based binding assays, SPR measurements) or with both binding partners free in solution (MST measurements)^21^. The results have shown that the affinity is strongly depending on the assay conditions but was generally found to be in the low micromolar to submicromolar range (668–1,350 nM for the full-length aptamer PA#2/8 and 95–522 nM for PA#2/8[S1-58]). If avidity effects can occur as seen in SPR experiments with immobilised full-length aptamer PA#2/8, a significant increase of the affinity to the low nanomolar range was observed (84–180 nM in comparison to 1,350 nM). Avidity effects are possible, because of the multivalent nature of Protein A. It provides more than one binding site for the aptamer, which seems to be overlapping with the known binding sites of immunoglobulins.

Comparing the results of the different binding assays, the ELONA setup used in this work allows a highly affine binding of aptamer PA#2/8 to Protein A. The formerly found increase of binding features after truncation to the variant PA#2/8[S1-58] was reproduced (Fig. 4).

### Applying Protein A-binding aptamer for targeting bacterial cells

The established assay conditions for ELONA were applied to evaluate the ability of aptamer PA#2/8 and PA#2/8[S1-58] to recognise and bind to Protein A in an intact cell context and so to target whole bacterial cells of *S. aureus*. Firstly, experiments were performed in which the microtiter plates were coated with suspensions of formaldehyde-fixed cells of *S. aureus,* and 5′- or 3′-biotinylated aptamer was added for binding. Starting from a cell suspension with an OD_600nm_ of 0.7 four dilution steps of 1:5, 1:10, 1:30, and 1:100 were prepared and used for coating. Two cell types were chosen because of their difference in Protein A expression. The *S. aureus* Cowan strain (CS) is known as a highly Protein A-producing strain, in contrast to the Protein A-deficient Wood46 strain (WS). Formaldehyde-fixed cells of both strains are commercially available and were prepared by a method ensuring binding of IgG. Protein A is well known for its interaction with the Fc regions of immunoglobulins, especially of several subclasses of human IgG and of IgG from other mammalian species^31^,^32^. Therefore, biotinylated human IgG was used as binding reagent to assess the successful immobilisation of *S. aureus* cells in microtiter plates. As expected, high signals were observed for binding of IgG to *S. aureus* CS, which stepwise decreased following the dilution of the cell suspensions used for coating (Fig. 5). In contrast, the binding of IgG to *S. aureus* WS was significantly lower, whereas only background binding signals were observed for the negative control *E. coli* K12 (living cells). Such differentiation between both cell types of *S. aureus* was also observed with aptamer PA#2/8 and PA#2/8[S1-58] as binding reagent, especially if a high cell density (cell suspensions with an OD_600_ = 0.7) was used for coating (Fig. 5). This clearly indicates the specific recognition and binding ability of the aptamers to the whole bacterial cells of *S. aureus* CS. Interactions of aptamer with cells of *S. aureus* WS resulted in lower signals comparable with those from interactions with living cells of *E. coli* K12, which therefore represent the range of unspecific background signals for the aptamers. The highest binding signal was measured for the 3′-biotinylated aptamer variant PA#2/8[S1-58]. But in contrast to IgG, the signal intensity of aptamer binding generally went rapidly down already with the first dilution step (1:5) of the cell suspension used for coating. Only background signals were measured for the negative controls using the unselected library or the truncated aptamer variant PA#2/8[S1-50]. For the latter has previously been shown that it is non-functional in ELONA (Fig. 3).

Experiments with the same ELONA setup were performed in which the microtiter plates were coated with living cell suspensions of two hospital-acquired isolates of *S. aureus* 96–01678 and 05–01042^33^. Biotinylated IgG as positive control for cell immobilisation and the best binding aptamer variant PA#2/8[S1-58] (3′Bio) were applied for interaction with immobilised cells (Fig. 6). The binding signals of IgG correlate with the amount of cells used for coating the microtiter plates. Very high signals were obtained for interaction of IgG with *S. aureus* isolate 05–01042. Significantly lower signals were measured for interaction with *S. aureus* isolate 96–01678. The results for the formalin-fixed cells of *S. aureus* CS were similar to those shown in Fig. 5. The obvious differences in the binding ability of IgG to the *S. aureus* isolates suggest differences in the Protein A content of both bacterial strains. This finding was also reflected by using aptamer PA#2/8[S1-58] as binding reagent. Remarkably high signals were obtained for interaction of the aptamer to *S. aureus* isolate 05–01042, which stepwise decreased following the dilution of the cell suspensions used for coating and reached the range of background signals between cell dilutions of 1:30 and 1:100 (corresponding to OD_600_ = 0.023 and 0.007). In contrast, the binding results of the aptamer to *S. aureus* isolate 96–01678 were similar to those obtained for its interaction with formalin-fixed *S. aureus* CS.

The ELONA experiments demonstrate the ability of aptamer PA#2/8 and the truncated variant PA#2/8[S1-58] to detect Protein A-expressing intact cells of the pathogenic bacterium *S. aureus*. They can distinguish between cells with different Protein A content and availability on the cell surface. The full-length aptamer was originally selected for the purified target Protein A by FluMag-SELEX^21^,^34^, but it is also able to recognise and bind to Protein A located on the cell surface. The first aptamers for *S. aureus* were developed by Cao *et al*. using a whole bacterial cell-based SELEX procedure^35^. They selected a set of DNA aptamers for the specific detection of *S. aureus* targeting different cell surface structures as shown by competition experiments. They also could demonstrate an enhanced binding capacity and improved sensitivity of a combination of five suitable aptamers in comparison to the individual ones. Further studies also focused on DNA aptamer developments for *S. aureus* using killed or living bacterial cells as complex selection target^36^,^37^,^38^. However, none of these studies identified the specific molecular targets on the cell surface, which are recognised by the selected aptamers. Other than the cell-based SELEX procedure, the use of a defined surface structure of bacterial cells as target also represents a successful aptamer selection strategy. By this way, DNA aptamers were selected for peptidoglycan, one of the main components of the bacterial cell wall^39^, and RNA aptamers were developed using teichoic acid as specific target of the gram positive bacterial cell wall of *S. aureus*^40^,^41^. Two further studies also reported the generation of aptamers for Protein A^42^,^43^. Baumstummler *et al*. have selected special modified DNA aptamers, so called SOMAmers, possessing chemical features that enhance the specificity and affinity of protein-nucleic acid interactions^42^. Dissociation constants in the subnanomolar range were measured for their binding to Protein A. Friedman *et al*. recently described the direct selection of RNA aptamers from a special 2′-fully modified RNA library for Protein A as a model target^43^. They could demonstrate the high nuclease and serum stability of these aptamers, which were able to bind to purified Protein A with affinities in the middle to low nanomolar range. All of these aptamers selected for specific molecular targets of the cell surface of *S. aureus* were shown to be also functional for the recognition and binding of the intact bacterial cells.

The main purpose of these aptamer developments was to obtain analytical and diagnostic tools suitable for the specific recognition and rapid detection of the bacterial pathogen *S. aureus*. The functionality of these aptamers was shown applying different detection methodologies, like microscopic methods, flow cytometry, real-time PCR, radiolabelling or fluorescence-based methods, or the use of aptamer-functionalised nanoparticles^35^,^36^,^37^,^38^,^39^,^40^,^41^,^42^,^43^,^44^,^45^,^46^. The development of special designed aptamer-based bioassays and aptasensors with focus on improved sensitivity is of great interest to lower the detection limit for the bacterial cells of *S. aureus*. Examples are the potentiometric biosensor based on single-walled carbon nanotubes with a detection limit of 8 × 10^2^ cfu/ml^47^, colorimetric microplate assays based on aptamer recognition coupled with tyramide signal amplification (TSA) technology with a detection limit of 8–9 cfu/ml^19^,^20^, or luminescent bioassays based on aptamer-functionalised magnetic nanoparticles combined with upconversion nanoparticles able to detect 25 cfu/ml^48^. Moreover, the limit of detection could be lowered down to 1 cfu/ml of *S. aureus* using a graphene-based potentiometric biosensor^49^.

The recently selected aptamer PA#2/8^21^ used in this study expands the portfolio of the known *S. aureus* targeting aptamers. After a detailed characterisation of the interaction between aptamer and Protein A by different methods^21^, ELONA was initially used to show the ability of this aptamer to recognise Protein A on the cell surface of *S. aureus*. But there are some conditions, which need to be improved especially for a stable and sensitive detection of the bacterial cells. Therefore, future work will focus on optimisation of the direct immobilisation of bacterial cells in microtiter plates or on integrating the aptamer in a sandwich ELONA format. Other assay formats will be more suitable for analytical applications, for instance aptamer-based biosensors (aptasensors) using the aptamer as the bio-recognition element, and should be tested in particular with regard to assay sensitivity. Different sensing principles are possible by use of electrochemical (amperometric, impedimetric), optical or surface acoustic wave transducers. Coupling the aptamer with electrochemically or optically active markers is very easy. Applicability of the aptamer in flow cytometry is feasible as well.

### CD spectroscopic and electrophoretic analyses of Protein A-binding aptamer

CD spectroscopy was used to investigate the structural features of the Protein A-binding aptamer PA#2/8, especially concerning its ability to fold into a G-quadruplex structure. The method also gives the possibility to analyse the binding complex and compare it with the sum of aptamer and Protein A to confirm the interaction between both. For this reason, all the studied aptamers, both recombinant and native Protein A, and the aptamer-Protein A-complex were analysed separately. The difference spectrum between aptamer-protein-complex and arithmetic sum of both the aptamer and the protein represents a residual interaction between these two molecules (Fig. 7a). Clear difference in the wavelength range downstream of 240 nm was observed, which represents a signature of certain interaction between PA#2/8 and native Protein A. The truncated aptamer variants PA#2/8[S1-58], PA#2/8[S1-50] and PA#2/8[S1-43] also show similar features. However, the truncated variant PA#2/8[S28-58] with an additional removing of the first 27 nucleotides at the 5′-end (Table 1) resulting in loss of function does not show any difference signal downstream of 240 nm. This G-rich oligonucleotide was used as negative control because the clear positive signal at 265 nm obtained with circular dichroism shows that it also forms G-quadruplex.

Results in Fig. 7b,c represent CD spectra and melting curves of the full-length aptamer PA#2/8 and its 3′-truncated variants (PA#2/8[S1-58], PA#2/8[S1-50], PA#2/8[S1-43]). There is a clear demonstration that each of the aptamers shows a positive signal near 265 nm and a negative signal near 240 nm (Fig. 7b). This is typical for parallel G-quadruplexes in which most of guanines occur in an *anti-*conformation^50^. The relative molar CD intensity for PA#2/8 is 1. The intensities of shortened aptamers are a little lower than observed for the full-length aptamer. However, just for comparison, the molar CD intensity of known c-kit and c-myc G-quadruplexes is less than a half of that obtained for PA#2/8[S1-58] or PA#2/8[S1-43] in case we use the comparison described recently^51^. Therefore, it can be assumed that the Protein A-binding aptamer variants predominantly form di- or multimeric G-quadruplexes. The monomeric structure may be formed mainly by PA#2/8[S1-43]. In this case, only two G-cluster of 5 guanines are present, which can also form a two-layered G-quadruplex with two intervening loops of one guanine and a longer central loop or a three-layered, broken-stranded G-quadruplex^50^,^52^,^53^.

The melting curves of the aptamer variants in Fig. 7c were obtained at 265 nm, where data of CD signals in a temperature range from 20 °C to 110 °C were collected. Typical melting profiles show a sigmoidal dependence of the melting process representing a two-state equilibrium between the folded and unfolded state of the aptamers. Such S-shaped melting curve was obtained for the aptamer variant PA#2/8[S1-58]. A melting temperature of 73 °C was determined. The melting curves of the other aptamer variants clearly deviate from a sigmoidal curve. This means the melting process for these aptamers is not governed by a two-state mechanism. Nevertheless, all of these studied aptamer variants are very stable because the melting process starts at temperatures higher than 60 °C and is not still finished at 90 °C. Based on the non-sigmoidal shape of the melting curves the co-existence of many different G-quadruplex topologies in solution with different melting behaviours is proposed.

Electrophoretic separation can provide valuable information about the molecularity of a G-quadruplex structure and the potential to form multimeric structures^51^. The electrophoretic analysis was performed with the full-length aptamer and its 3′-truncated variants under the same conditions as used for CD measurements. Different unstructured DNA oligonucleotides (d(AC)_9_, d(AC)_14_, d(AC)_18_, d(AC)_26_, d(AC)_36_) and two known G-quadruplex forming oligonucleotides d(G_3_T_2_A)_3_G_3_) and d(G_3_T_2_A)_7_G_3_T_14_ were used as molecular standards. The electrophoretic pattern of the shortest aptamer variant PA#2/8[S1-43] is dominated by a fast moving DNA band in a size range lower than expected from the sequence length and from the comparison with the unfolded standards (Fig. 7d). This observation is typical for compact structured sequences including intramolecular G-quadruplexes. Comparable fast moving bands are also present in the electrophoretic patterns of the other three aptamer variants but with considerably lower intensity. More slowly moving bands can also be identified in the gel in a size range that would indicate a more complexed structure formed by at least two aptamer molecules. The smear observed between the faster and slower moving bands can be explained by the presence of transient conformers, which represent intermediates between at least two different topological states during electrophoretic separation. A higher population of such transient structures was found for the variant PA#2/8[S1-50], which can be the cause of its lower binding ability to Protein A. In addition, high-ordered or multimeric structures seem to be the prevalent forms especially for the full-length aptamer PA#2/8 as indicated by the stained material near the gel origin, which was not separated under the applied electrophoretic conditions (Fig. 7d, lane 1). Monomeric and dimeric structures are barely visible. High-ordered structures were also found for the truncated aptamer variants PA#2/8[S1-58] and PA#2/8[S1-50], but not for PA#2/8[S1-43] (Fig. 7d, lanes 2–4). The results of electrophoretic analysis give an impression of the polymorphic nature of the aptamer. Although the Protein A-binding functionality remains after 3′-truncation, the preferred aptamer folding topology is changed.

The folding of the Protein A-binding aptamer seems to be highly complex. Two sequence regions of the aptamer could be identified to be involved in its target-binding function. The sequence region at the 5′-end (including the primer binding site) is able to form a protruding imperfect hairpin. This secondary stem-loop motif was predicted by a secondary structure analysis using the free-energy minimisation algorithm of the web based tool mfold with salt correction (Supplementary Fig. S1)^21^,^54^,^55^,^56^. However, tertiary conformations of DNA like G-quadruplexes cannot be predicted by mfold. The stem-loop motif is build up by 5–7 nt forming the imperfect stem and 1–5 nt forming the loop. Results of previously described aptamer truncation experiments clearly show that this sequence motif is essential for binding of the aptamer to Protein A^21^. Removing it leads to the complete loss of function. On the other hand, the G-quadruplex forming intern region of the aptamer also contributes to its function. Further shortening of the minimal G-quadruplex forming motif of the variant PA#2/8[S1-43] by removing the G-cluster next to the 3′-end also leads to the loss of function.

Both of these sequence regions have also the potential to build up dimeric up to multimeric structures. Dimerisation is possible based on the inverted repeat near the 5′-end of the aptamer. The result is a dimer consisting of an imperfect double-stranded region with at least twelve Watson-Crick base-pairs between the 5′-ends of two aptamer molecules. A second mode of dimerisation is conceivable concerning the G-quadruplex motif. Especially in case of the longer aptamer variants PA#2/8[S1-50] and PA#2/8[S1-58] and the full-length aptamer PA#2/8, it is very likely that they also form dimeric and multimeric structures by stacking and interlocking of two or more G-quadruplex units. Such types of higher-order structures could explain the electrophoretic results and a significantly higher elliptic CD signal, which was observed^51^.

However at this stage of investigation, a definitive answer to the aptameric structure during interaction with Protein A cannot be given. Further examinations by methods like NMR or X-ray crystallography could provide more significant details about structural elements that are critical to a stable aptamer folding and/or that are directly involved in interaction with the target protein.

## Conclusions

The aptamer PA#2/8 was selected for Protein A by the FluMag-SELEX process, and its binding features to the target protein were intensively investigated by different methods (SPR, MST, bead-based assays)^21^. The main objective of the current study was to investigate the ability of the aptamer to recognise intact bacterial cells of *S. aureus*, which express Protein A on their cell surfaces.

An aptamer-based Enzyme-Linked OligoNucleotide Assay (ELONA) was established and the functionality of the assay was shown firstly with Protein A as the original aptamer selection target. The aptamer strongly binds to native and recombinant Protein A and clearly distinguishes its target Protein A from functional related proteins as well as unspecific control proteins. PA#2/8 and its truncated variant PA#2/8[S1-58] can be applied as 5′- or 3′-biotinylated aptamer in ELONA. But in contrast to previous results using bead-based binding assays and MST^21^, the further shortened variants PA#2/8[S1-50] and PA#2/8[S1-43] were not functional. ELONA allows a highly affine detection of Protein A by the aptamer. In general, the truncated 3′-biotinylated aptamer PA#2/8[S1-58] shows the best binding features with an affinity constant of K_D_ = 11.3 ± 1.4 nM.

Applying intact bacterial cells of *S. aureus* in ELONA, the results clearly demonstrate the specific recognition and binding of the aptamer to the cells via Protein A. PA#2/8 and its truncated variant PA#2/8[S1-58] were able to differentiate between the highly Protein A-producing *S. aureus* Cowan strain and the Protein A-deficient Wood46 strain (both as formaldehyde-fixed cells) or living *E. coli* as the negative control. Two hospital-acquired isolates were applied as living *S. aureus* cells in ELONA in combination with the best binding aptamer variant PA#2/8[S1-58] (3′Bio). Strong differences in the binding signal to the isolates were obtained, with a remarkable high signal to one of them, indicating a different Protein A content of both.

The basic ELONA works well but should be optimised for improved binding results. In particular, the development of a sandwich assay or competitive assay could avoid the immobilisation of the target cells and improve the assay handling. More important for examination of a potential analytical application of the aptamer is its integration in analytical systems rigorously aimed at sensitivity, robustness, and practicality.

Because folding into a specific structure is an essential characteristic of each aptamer, structural features strongly influence the choice of a special assay design and detection principle to retain the aptameric function and to ensure a sensitive interaction with the target. For the Protein A-binding aptamer, two structural elements seem to be essential for its function. Previous studies have shown that a free 5′-end of the aptamer sequence, which is able to fold into an imperfect stem-loop motif, is crucial for binding to Protein A, whereas the 3′-end can be truncated^21^. The intern aptamer sequence contains four differently sized G-stretches, which indicate a G-quadruplex structure. This could be demonstrated in the current study. The full-length aptamer and also its 3′-truncated variants are able to fold into parallel G-quadruplex structures. This also applies for the shortest functional variant containing only two G-stretches with five guanines each (PA#2/8[S1-43]). The combination of both structural elements is important for the functionality of the aptamer. Removing one of them leads to the loss of function. Moreover, the results also reveal a highly polymorphic characteristic of the aptamer. Beside monomeric structures, dimeric or multimeric structures seem to be possible with the involvement of different G-quadruplex topologies. More specialised methods like NMR or X-ray crystallography are necessary to give an answer about the mode of interaction between aptamer and Protein A and the preferred binding structure of the aptamer.

## Methods

### Proteins and buffer solutions

Native Protein A from *Staphylococcus aureus* (P3838, P6031), recombinant Protein A (P7837, expressed in *E. coli*), and human serum albumin (HSA, A9511) were purchased from Sigma-Aldrich (Germany). Human immunoglobulin G (IgG) biotin conjugated (Rockland 009–0602) was from BIOTREND Chemikalien GmbH (Germany). Recombinant Protein G and Protein L (Pierce 21193 and 21189, expressed in *E. coli*) were purchased from Fisher Scientific (Germany).

Phosphate-buffered saline was prepared for use in ELONA experiments (PBS; 137 mM NaCl, 2.7 mM KCl, 1.47 mM KH_2_PO_4_, and 8.1 mM Na_2_HPO_4_, pH 7.4).

### DNA oligonucleotides

All aptamers and oligonucleotides used in this study are listed in Table 1. Aptamer PA#2/8 was selected for Protein A from *Staphylococcus aureus* by the FluMag-SELEX process as described previously^21^. Three functional 3′-truncated variants (PA#2/8[S1-58], PA#2/8[S1-50], and PA#2/8[S1-43]) of the full-length aptamer were also included in this study. BANK-C represents the unselected SELEX library and was used as negative control. This library consists of a multitude of different oligonucleotides. Each of them contains a central random region of 40 nt (N) flanked by specific sequences of 18 nt at the 5′- and 3′-end. Biotin conjugated aptamers and BANK-C were synthesised by BioSpring GmbH (Germany) as 5′- or 3′-biotinylated oligonucleotides (indicated with 5′Bio or 3′Bio). Unmodified aptamers and derived oligonucleotides for CD and electrophoretic analyses were purchased from Metabion GmbH (Germany).

### Bacterial strains and cultivation

Formaldehyde-fixed cells of *Staphylococcus aureus* Cowan strain (CS) (P7155; Protein A-producing strain; preparation method ensures binding of IgG) and Wood46 strain (WS) (S2014; Protein A-deficient strain; same preparation method as for P7155; binds less than 10% of the rabbit IgG bound by P7155 as declared by manufacturer) were obtained as crude cell suspension from Sigma-Aldrich (Germany). Two hospital-acquired *Staphylococcus aureus* isolates 96–01678 (09–03240; *spa* type t032, MRSA) and 05–01042 (*spa* type t030, MRSA) were provided by the National Reference Centre for Staphylococci and Enterococci at the Robert Koch Institute, Wernigerode Branch (Germany)^33^. *E. coli* K12 was given from the strain collection by the Helmholtz Centre for Environmental Research – UFZ, Leipzig (Germany).

*E. coli* K12 was grown overnight in 10 mL Luria-Bertani (LB) medium at 37 °C. 25 mL of fresh medium was inoculated with 5 mL overnight culture and cultivated again for 3 h at 37 °C. After that, 1 mL aliquots of cells were harvested by centrifugation at 10,000 g for 10 min, washed three times with 0.1 M sodium phosphate buffer pH 7.4, finally resuspended in 500 μL 0.1 M sodium phosphate buffer pH 7.4, and stored at 6–8 °C until use. A cell suspension with an OD_600_ of 0.7 was prepared and applied for ELONA.

*S. aureus* isolates 96–01678 and 05–01042 were grown on Müller-Hinton agar plates with 3% sheep blood. Cells of one colony were incubated overnight in 5 mL liquid culture at 37 °C and then used to inoculate 5 mL fresh medium for further cultivation at 37 °C for 5–5.5 h. *S. aureus* cells were harvested and washed as described above. Cells were diluted in 0.1 M sodium phosphate buffer pH 7.4 to an OD_600_ of 0.7 and used for ELONA.

### ELONA (Enzyme-Linked OligoNucleotide Assay)

**Figure 1:**
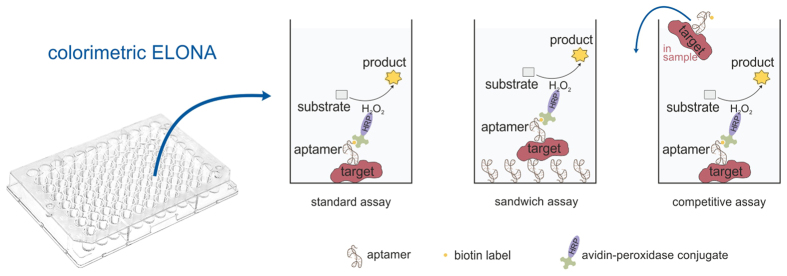
Schematic representation of different ELONA formats (Enzyme-Linked OligoNucleotide Assay) used for aptamer-based protein detection.

**Figure 2:**
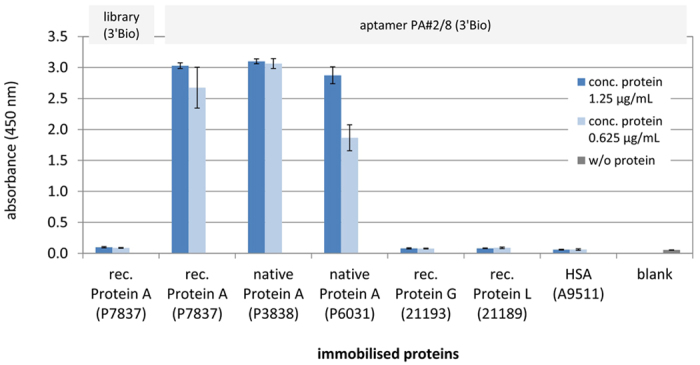
Evaluation of target binding of aptamer PA#2/8 and its specificity using ELONA. Protein solutions of 1.25 μg/mL and 0.625 μg/mL were used for coating the microtiter plates with different Protein A variants (native and recombinant), functional related proteins, and HSA. 100 nM of 3′-biotinylated aptamer PA#2/8 (3′Bio) was added for binding, whereas 500 nM of 3′-biotinylated, unselected SELEX library (3′Bio) was used as negative control. The blank reaction represents the control without any protein coating. The results of three separate experiments were averaged. Each of them was made with four replicates of each specific DNA-protein interaction (three replicates for blank reaction).

**Figure 3:**
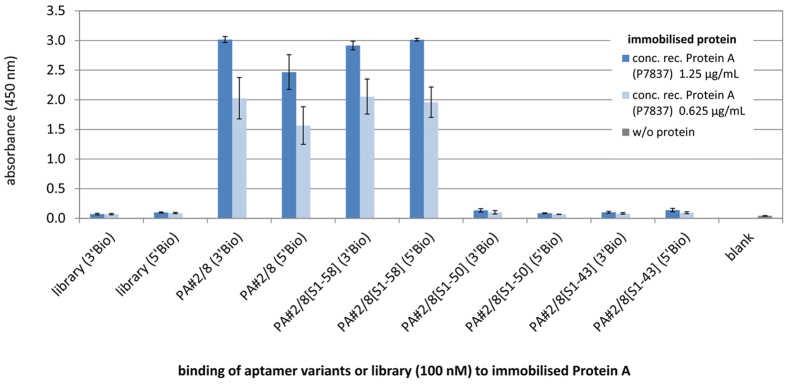
Evaluation of binding ability of three truncated aptamer variants to Protein A in comparison to the full-length aptamer PA#2/8 by ELONA. Two solutions of recombinant Protein A (P7837) with a concentration of 1.25 μg/mL and 0.625 μg/mL respectively were used for coating the microtiter plates. 100 nM of each biotinylated aptamer variant and the library as negative control were applied for binding. In addition, the effect of biotinylation site (5′Bio or 3′Bio) of the oligonucleotides was tested. The blank reaction represents the control without any protein coating. The results of three separate experiments were averaged, whereby each of them was made with four replicates of each specific DNA-protein interaction (three replicates for blank reaction).

**Figure 4:**
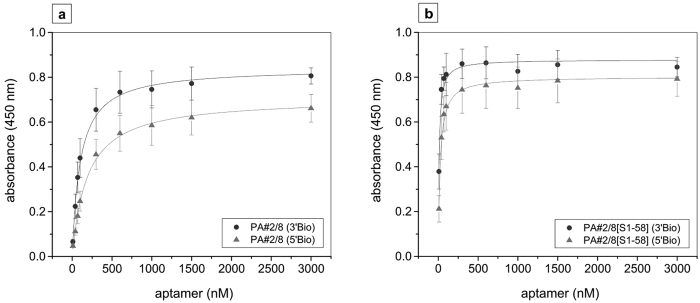
Assessment of binding affinity of aptamer PA#2/8 (**a**) und PA#2/8[S1-58] (**b**) to Protein A by ELONA. Microtiter plates were coated with 1.25 μg/mL recombinant Protein A (P7837), and a concentration series in the range of 10–3,000 nM of each 5′- or 3′-biotinylated aptamer (5′Bio or 3′Bio) was applied for binding. Each data point represents the averaged results of four separate experiments, each with two replicates.

**Figure 5:**
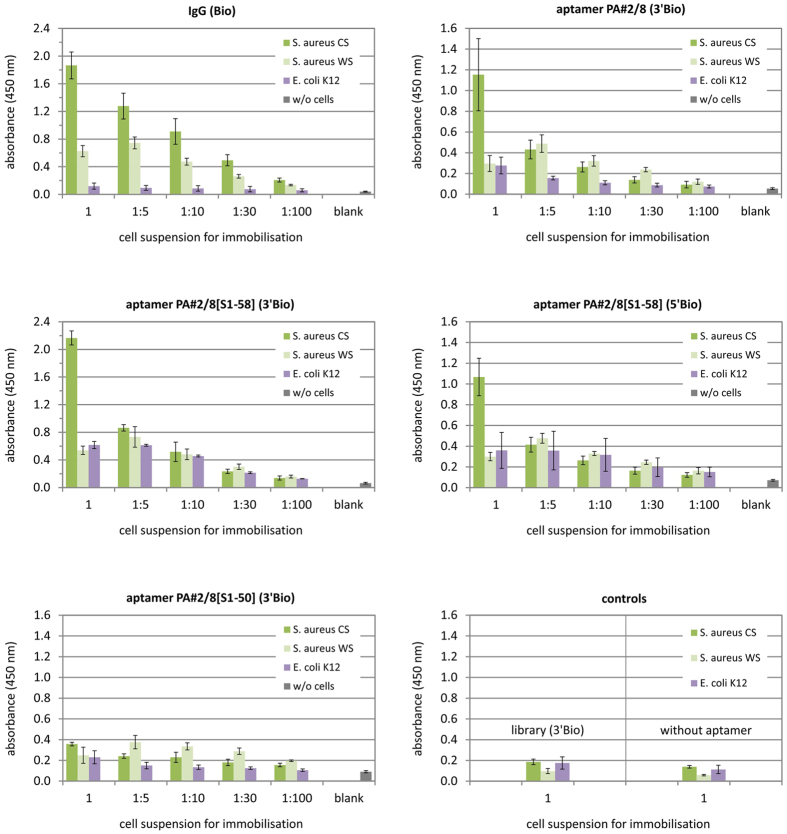
Evaluation of binding ability of aptamer PA#2/8 and PA#2/8[S1-58] to bacterial cells of *S. aureus* by ELONA. Formaldehyde-fixed cells of *S. aureus* CS (Protein A-producing Cowan strain) and *S. aureus* WS (Protein A-deficient Wood46 Strain) as well as living cells of *E. coli* K12 as negative control were used for coating the microtiter plates. Cell suspensions with an OD_600_ = 0.7 (1) and further stepwise dilutions (1:5, 1:10, 1:30, 1:100, corresponding to OD_600_ = 0.14, 0.07, 0.023, 0.007) were applied. 100 nM of each biotinylated aptamer variants was added, respectively, for binding. The blank reaction represents the assay control without any cell coating. A positive control for binding to immobilised cells is represented by adding of 0.13 nM biotinylated IgG. Negative controls include reactions with the library (3′Bio), non-functional aptamer variant PA#2/8[S1-50] (3′Bio), and without biotinylated aptamer or IgG. Averaged results are shown: 2–3 separate experiments per aptamer probe and 11 experiments with IgG, whereby each of them was made with 2–3 replicates of each specific interaction. All experiments contained 1 control reaction per cell type with the library and 3 control reactions per cell type without aptamer probes or IgG.

**Figure 6:**
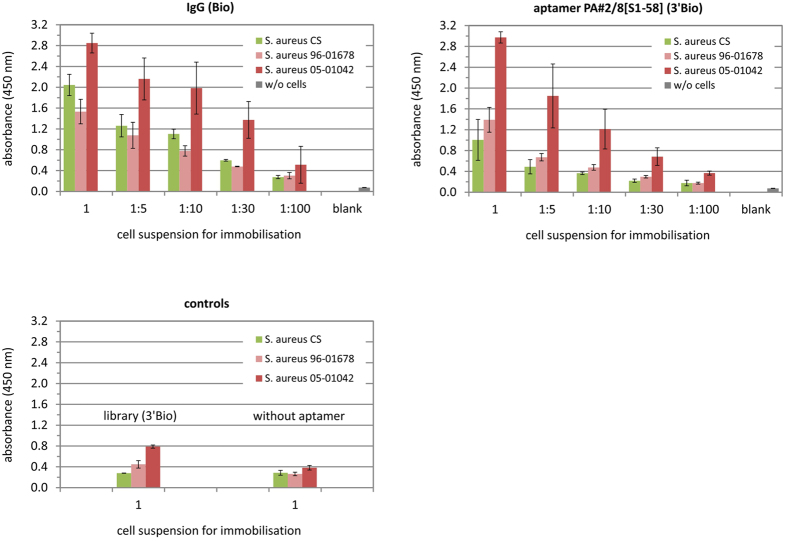
Binding ability of aptamer PA#2/8[S1-58] to living cells of *S. aureus*. Two *S. aureus* isolates 96–01678 and 05–01042 as well as formaldehyde-fixed cells of *S. aureus* CS (Protein A-producing Cowan strain) were used for coating the microtiter plates. Cell suspensions with an OD_600_ = 0.7 (1) and further stepwise dilutions (1:5, 1:10, 1:30, 1:100) were applied. 100 nM of PA#2/8[S1-58] (3′Bio) were added for binding in comparison to 0.13 nM IgG (Bio) as positive control for binding to immobilised cells. The blank reaction represents the assay control without any cell coating. Negative controls include reactions with the library (3′Bio) and without biotinylated aptamer or IgG. The results of 2 separate experiments were averaged, whereby each of them was made with 1 (IgG) or 2 (aptamer) replicates of each specific interaction. All experiments contained 1 control reaction per cell type with the library and 3 control reactions per cell type without aptamer or IgG binding reagent.

**Figure 7:**
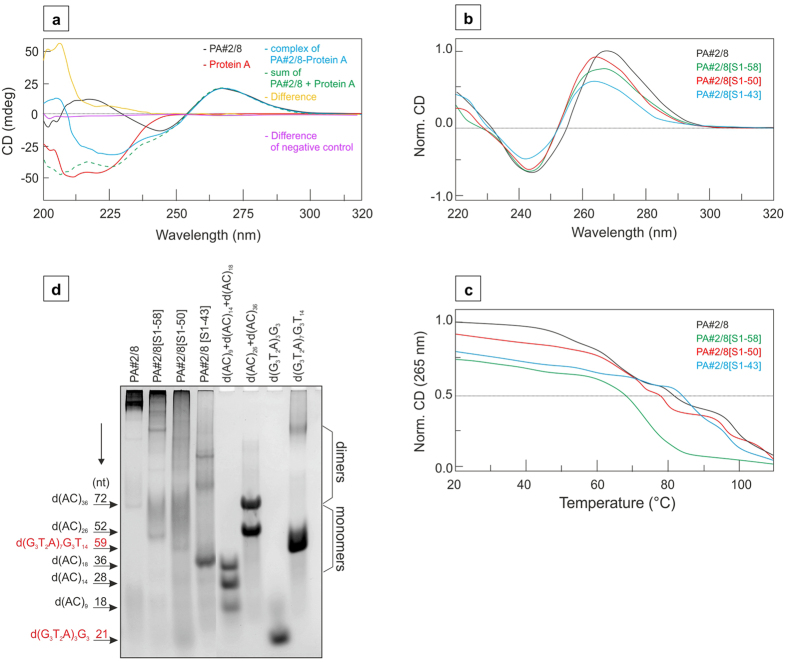
CD spectra of Protein A-binding aptamer variants and electrophoretic results. **(a)** The representative CD spectra of DNA aptamer PA#2/8 (350 nM), native Protein A (230 μg/mL) and aptamer-protein-complex (PA#2/8-Protein A) are represented by black, red and blue line, respectively. The arithmetic sum of PA#2/8 and Protein A is represented by a dashed green line. The difference spectrum between complex and arithmetic sum is represented by a yellow line. The aptamer variant PA#2/8[S28-50] truncated at both ends was used as negative control. In this case, the difference between arithmetic sum and complex shows no detectable signal (purple line). **(b)** CD spectra of different Protein A-binding aptamer variants in modified 25 mM Britton-Robinson buffer (pH 7.6) in the presence of 100 mM NaCl, 10 mM MgCl_2_, 1 mM CaCl_2_ and 5 mM KCl were compared (PA#2/8, black line; PA#2/8[S1-58], green line; PA#2/8[S1-50], red line and PA#2/8[S1-43], blue line). The aptamers were used at a concentration of 350 nM. **(c)** The corresponding CD melting curves obtained at 265 nm are depicted. **(d)** Separation of different aptamer variants by native gel electrophoresis and visualisation by StainsAll staining. The mobilities of molecular standards (mix of d(AC)_9_ + d(AC)_14_ + d(AC)_18_, mix of d(AC)_26_ + d(AC)_36_, d(G_3_T_2_A)_3_G_3_) and d(G_3_T_2_A)_7_G_3_T_14_) are shown on the gel at the right. The positions of the standards are additionally indicated as arrows on the left including their sizes in nt. G-quadruplex forming standards are highlighted in red. Electrophoretic separation was performed in a 10% polyacrylamide gel in 25 mM Britton-Robinson buffer (pH 7.6) and 100 mM NaCl, 10 mM MgCl_2_ and 5 mM KCl at 37 °C. The loading buffer also contained 1 mM CaCl_2_ and 0.005% Tween 20.

The ELISA-like assay ELONA^2^ (Fig. 1) was adapted for work with the DNA aptamer PA#2/8 and its truncated variants PA#2/8[S1-58], PA#2/8[S1-50], and PA#2/8[S1-43]. The binding of the aptamers to their target Protein A (native or recombinant) was evaluated and related proteins (Protein G, Protein L, and HSA) were introduced to access the specificity of the aptamers.

Proteins were diluted to 1.25 mg/L or 0.625 mg/L in 0.1 M sodium phosphate buffer pH 7.4 and 100 μL/well of these solutions were incubated overnight at 4 °C in a polystyrene 96-well microtiter plate (MaxiSorp^TM^; Th. Geyer, Germany) with mild shaking. For blank reactions, the wells were treated with buffer solution in this step without any protein addition. After coating, the wells were washed two times in 200 μL washing buffer (0.3 M NaCl/PBS containing 0.05% Tween 20), followed by three washing steps with 200 μL binding buffer (100 mM NaCl, 20 mM Tris-HCl pH 7.6, 10 mM MgCl_2_, 5 mM KCl, 1 mM CaCl_2_, 0.005% Tween 20). 100 μL of 5′- or 3′-biotinylated aptamers (5′Bio or 3′Bio) diluted to 100 nM in binding buffer were added to each well. The aptamers were previously denatured for 8 min at 90 °C, cooled for 10 min on ice, and kept at room temperature for 5 min before applying. The plate was incubated at 21 °C for 1 h with mild shaking for binding of the aptamers to the immobilised protein. The SELEX library BANK-C was used in the same way as negative control at concentrations of 100 nM or 500 nM. After binding reaction, the wells were washed three times in 200 μL binding buffer to remove unbound oligonucleotides. ExtrAvidin-peroxidase conjugate (E2886; Sigma-Aldrich, Germany) was diluted 1:10,000 in binding buffer to a concentration of 0.2–0.25 μg/mL and 100 μL were applied to each well, followed by an incubation at 21 °C for 1 h with mild shaking. The wells were washed again five times with 200 μL binding buffer. After that, 100 μL of 3,3′,5,5′-tetrametylbenzidine (TMB) substrate solution (Thermo Scientific Pierce 1-Step Ultra TMB-ELISA; Fisher Scientific, Germany) was added to each well and incubated for 15 min at room temperature in the dark. The reaction was stopped by addition of 50 μL/well of 0.5 M H_2_SO_4_ and the optical density was measured at 450 nm using the Wallac 1420 Victor^2^ V Multilabel Counter (PerkinElmer, Germany) and Infinite^®^ M1000 PRO (Tecan, Switzerland), respectively. As specified in the results and discussion section, the results of up to four separate experiments were generally averaged, whereby each experiment represents one microtiter plate and was made with one to four replicates of each specific interaction.

In another set of experiments, the Thermo Scientific Pierce 1-Step Slow TMB-ELISA (Fisher Scientific, Germany) was used as substrate solution, which is recommended as an ideal substrate for kinetic studies. To evaluate the affinity of the aptamers to Protein A, a protein solution of 1.25 mg/L was used for coating the wells and biotinylated aptamers were applied in a concentration series of 10–3,000 nM for binding. On the basis of the measured absorbance at 450 nm, saturation curves were obtained and the dissociation constants K_D_ were calculated by non-linear regression analysis using OriginPro 9.0 (OriginLab Corporation, Northampton, MA, USA).

Similar experiments were performed by coating the wells with bacterial cells of *Staphylococcus aureus* to assess the binding ability of aptamer PA#2/8 and truncated variants to Protein A in an intact cell context. The following cells were used: formaldehyde-fixed cells of the Protein A-producing *S. aureus* Cowan strain (CS) and of the Protein A-deficient *S. aureus* Wood46 Strain (WS), living cells of *S. aureus* isolates 96–01678 and 05–01042^33^, as well as living cells of *E. coli* K12 as negative control. 100 μL/well from cell suspensions with an OD_600_ of 0.7 and from further diluted suspensions in 0.1 M sodium phosphate buffer pH 7.4 (1:5, 1:10, 1:30, and 1:100, corresponding to OD_600_ = 0.14, 0.07, 0.023, and 0.007) were coated onto microtiter plates overnight at 4 °C with mild shaking. The next steps were the same as described above. In addition, 100 μL of 0.13 nM biotinylated IgG in binding buffer instead of biotinylated aptamers was used as positive control for binding to immobilised *S. aureus* cells.

### Circular Dichroism study

Circular Dichroism (CD) spectra were recorded on a Jasco J-810 spectropolarimeter (Easton, MD, USA) equipped with a PTC-423L temperature controller using a quartz cell of 1-mm optical path length in a reaction volume of 150 μL. All the other parameters and conditions were the same as those which were described previously^53^,^57^. The modified Britton-Robinson buffer was used in all spectral measurements where TRIS was used instead of KOH (NaOH): 25 mM phosphoric acid, 25 mM boric acid, and 5 mM acetic acid, supplemented by 100 mM NaCl, pH 7.6, 10 mM MgCl_2_, 5 mM KCl, 1 mM CaCl_2_; pH was adjusted by TRIS to a final value of 7.6. This buffer is also suitable for the electrophoresis.

### CD melting curves

CD melting profiles were collected at 265 nm. The temperature ranged from 10 to 110 °C, and the heating rate was 0.25 °C per min. The melting temperature (T_m_) was defined as the temperature of the mid-transition point between two states. T_m_ was estimated from the peak value of the first derivative of the fitted curve^57^.

### Electrophoresis

Native polyacrylamide gel electrophoresis (PAGE) was performed in a temperature-controlled vertical electrophoretic apparatus (Z375039-1EA; Sigma-Aldrich, San Francisco, CA). Gel concentration was 10% (19:1 monomer to bis ratio, AppliChem, Darmstadt). Approximately 2 μg of sample was loaded onto 14 × 16 × 0.1 cm gels. Electrophoreses were performed at 37 °C for 3 h at 126 V (~8 V cm^−1^). DNA oligomers were visualised with Stains-all immediately after the electrophoresis, and the electrophoretic record was photographed on a white pad with a Nikon D3100 camera. Additional staining with silver was performed consequently. The modified Britton-Robinson buffer was used in all electrophoretic separations; pH was adjusted by TRIS to a final value of 7.6.

## Additional Information

**How to cite this article**: Stoltenburg, R. *et al*. G-quadruplex aptamer targeting Protein A and its capability to detect *Staphylococcus aureus* demonstrated by ELONA. *Sci. Rep.* **6**, 33812; doi: 10.1038/srep33812 (2016).

## Supplementary Material

Supplementary Information

## Figures and Tables

**Table 1:** Aptamers and oligonucleotides applied in this study.

Oligonucleotide	Sequence (5′ → 3′)	Length
full-length aptamer		
PA#2/8	ATACCAGCTTATTCAATTAGCAACATGA**GGGGG**ATAGA**GGGGG**T**GGG**TTCTCTC**GG**CTACAATCGTAATCAGTTAG	76nt
truncated aptamer variants		
PA#2/8[S1-58]	ATACCAGCTTATTCAATTAGCAACATGA**GGGGG**ATAGA**GGGGG**T**GGG**TTCTCTC**GG**CT	58nt
PA#2/8[S1-50]	ATACCAGCTTATTCAATTAGCAACATGA**GGGGG**ATAGA**GGGGG**T**GGG**TTC	50nt
PA#2/8[S1-43]	ATACCAGCTTATTCAATTAGCAACATGA**GGGGG**ATAGA**GGGGG**	43nt
other oligonucleotides		
PA#2/8[S28-50]	A**GGGGG**ATAGA**GGGGG**T**GGG**TTC	23nt
BANK-C	ATACCAGCTTATTCAATT-------------------N_40_-------------------ACAATCGTAATCAGTTAG	76nt

Fixed sequences at the 5′- and 3′-end of the aptamers and the SELEX library BANK-C (primer binding sites) are underlined. The G-stretches in the internal sequence region of the aptamers are shown in bold type (secondary structures predicted by mfold are shown in Supplementary Fig. S1).
