# Supplementary material for: G-quadruplex aptamer targeting Protein A and its capability to detect Staphylococcus aureus demonstrated by ELONA
Source: Sci Rep. 2016 Sep 21;6:33812. doi: 10.1038/srep33812 (PMC5030626; doi:10.1038/srep33812)
Supplement: Supplementary Information [file srep33812-s1.pdf]

Supplementary Information

G-quadruplex aptamer targeting Protein A and its capability to detect *Staphylococcus aureus* demonstrated by ELONA

Regina Stoltenburg[1]*, Petra Krafčiková[2], Viktor Víglaský[2], Beate Strehlitz[3]

[1]UFZ - Helmholtz Centre for Environmental Research, Department of Soil Ecology, Theodor-Lieser-Straße 4, 06120 Halle, Germany

[2]P.J.Šafárik University, Faculty of Sciences, Institute of Chemistry, Dept. of Biochemistry, Moyzesova 11, 04011 Košice, Slovakia

[3]UFZ - Helmholtz Centre for Environmental Research, Department Environmental and Biotechnology Centre, Permoserstraße 15, 04318 Leipzig, Germany

*Corresponding author (email: regina.stoltenburg@ufz.de)

Methods

Bead-based binding assay

Superparamagnetic Dynabeads® M-270 Streptavidin (Strep-MB) were purchased from Invitrogen/Life Technologies (USA). According to the manufacturer's instructions, biotinylated Protein A (P2165, Sigma-Aldrich, Germany) was immobilised on these streptavidin-coated magnetic beads to obtain Protein A-modified Strep-MB (Protein A/Strep-MB). 5'-fluorescein-labelled aptamer PA#2/8 and its truncated variants PA#2/8[S1-58], PA#2/8[S1-50], PA#2/8[S1-43], PA#2/8[S1-35] (5'-ATACCAGCTTATTCAATTAGCAACATGAGGGGGAT-3'), and PA#2/8[S19-76] (5'-AGCAACATGAGGGGGATAGAGGGGGTGGGTTCTCTCGGCTACAATC GTAATCAGTTAG-3') were synthesised by Microsynth (Switzerland) including a PAGE purification step. In addition to the aptamers, the SELEX library BANK-C was used as negative control. The 5'-fluorescein-labeled ssDNA from this library was prepared by PCR and PAGE purification as previously described[1].

For each individual assay, a fresh aliquot of 3×10^7 Protein A/Strep-MB was washed three times with 250 µL binding buffer (100 mM NaCl, 20 mM Tris-HCl pH 7.6, 10 mM $MgCl_2$, 5 mM KCl, 1 mM $CaCl_2$, 0.005% Tween 20). During the second washing step the beads were incubated at 21 °C for 5 min in binding buffer with mild shaking before magnetic separation of the beads. In parallel, 55 pmol fluorescein-labelled aptamer in 250 µL binding buffer were heated to 90 °C for 8 min, immediately cooled, and kept at 4 °C for 10 min followed by a short incubation at room temperature before adding it to the washed beads. Incubation was performed at 21 °C for 30 min with mild shaking for binding of the aptamers to Protein A/Strep-MB. Unbound aptamers were removed by 3 to 5 washing steps with 250 µL binding buffer and bound aptamers were then eluted twice by incubating the binding complexes in 250 µL binding buffer at 95 °C for 10 min with mild shaking. The amount of aptamers eluted from the beads was determined by fluorescence detection and calculation using a calibration curve.

The fluorescence measurements were performed in black 96-well microtiter plates from NUNC/Thermo Fisher Scientific (Germany) with a sample volume of 100 µL/well on a Wallac 1420 Victor^2 V Multilabel Counter (PerkinElmer, Germany) with excitation at 485 nm and emission at 535 nm (prompt fluorometry, time 1 s, CW-lamp energy 22500). Calibration curves were prepared in the range of 0.4 - 80 pmol/mL of fluorescein-labelled ssDNA from each aptamer variant and BANK-C.

Secondary structure prediction

The secondary structure analysis was performed by means of the free-energy minimisation algorithm according to Zuker[2] using the internet tool mfold at 21 °C with 100 mM [Na^+] and 10 mM [Mg^{2+}] (available at: http://mfold.rna.albany.edu/?q=mfold)[3,4].

Supplementary Figures

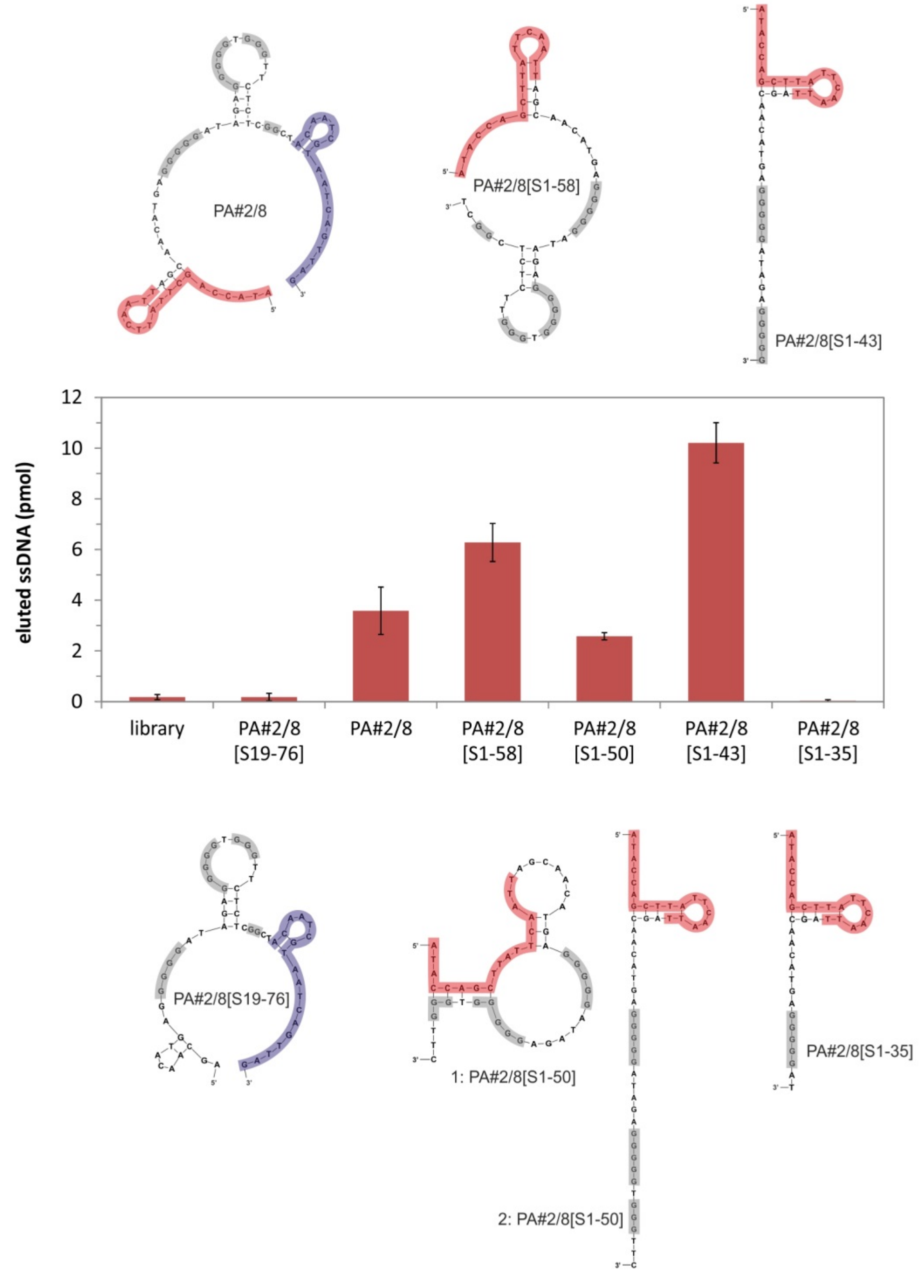

Supplementary Figure S1: Binding behaviour of the full-length aptamer PA#2/8 and truncated aptamer variants to Protein A using bead-based binding assays. Fluorescein-labelled aptamers were applied to Protein A/Strep-MB for binding. Target-bound aptamers were eluted and quantified. The SELEX library was used as negative control. In addition, the potential secondary structure of each aptamer variant is given. The primer binding sites (18 nt each) at the 5'- and 3'-end are highlighted in red and blue, respectively. The four G-stretches in the intern sequence region are highlighted in grey.

References

1. Stoltenburg, R., Schubert, T. & Strehlitz, B. In vitro Selection and Interaction Studies of a DNA Aptamer Targeting Protein A. *PLoS ONE* **10**, e0134403 (2015).

2. Zuker, M. Mfold web server for nucleic acid folding and hybridization prediction. *Nucleic Acids Res.* **31**, 3406-3415 (2003).

3. Peyret, N. *Prediction of nucleic acid hybridization: parameters and algorithms*, Wayne State University, Department of Chemistry, (2000).

4. SantaLucia, J., Jr. A unified view of polymer, dumbbell, and oligonucleotide DNA nearest-neighbor thermodynamics. *Proc. Natl. Acad. Sci. USA* **95**, 1460-1465 (1998).
